# ZYG11A Is Expressed in Epithelial Ovarian Cancer and Correlates With Low Grade Disease

**DOI:** 10.3389/fendo.2021.688104

**Published:** 2021-06-18

**Authors:** Laris Achlaug, Lina Somri-Gannam, Shilhav Meisel-Sharon, Rive Sarfstein, Manisha Dixit, Shoshana Yakar, Mordechai Hallak, Zvi Laron, Haim Werner, Ilan Bruchim

**Affiliations:** ^1^ Department of Human Molecular Genetics and Biochemistry, Sackler School of Medicine, Tel Aviv University, Tel Aviv, Israel; ^2^ Gynecology Oncology Laboratory, Department of Obstetrics and Gynecology, Hillel Yaffe Medical Center, Hadera, Israel; ^3^ The Ruth and Bruce Rappaport Faculty of Medicine, Technion – Israel Institute of Technology, Haifa, Israel; ^4^ David B. Kriser Dental Center, Department of Basic Science and Craniofacial Biology, New York University College of Dentistry, New York, NY, United States; ^5^ Endocrine and Diabetes Research Unit, Schneider Children’s Medical Center, Petah Tikva, Israel

**Keywords:** ZYG11A, insulin-like growth factor-1 (IGF1), IGF1 receptor, p53, ovarian cancer

## Abstract

The insulin-like growth factors (IGF) are important players in the development of gynecological malignancies, including epithelial ovarian cancer (EOC). The identification of biomarkers that can help in the diagnosis and scoring of EOC patients is of fundamental importance in clinical oncology. We have recently identified the *ZYG11A* gene as a new candidate target of IGF1 action. The aim of the present study was to evaluate the expression of ZYG11A in EOC patients and to correlate its pattern of expression with histological grade and pathological stage. Furthermore, and in view of previous analyses showing an interplay between ZYG11A, p53 and the IGF1 receptor (IGF1R), we assessed a potential coordinated expression of these proteins in EOC. In addition, *zyg11a* expression was assessed in ovaries and uteri of growth hormone receptor (GHR) knock-out mice. Tissue microarray analysis was conducted on 36 patients with EOC and expression of ZYG11A, IGF1R and p53 was assessed by immunohistochemistry. Expression levels were correlated with clinical parameters. qPCR was employed to assess zyg11a mRNA levels in mice tissues. Our analyses provide evidence of reduced ZYG11A expression in high grade tumors, consistent with a putative tumor suppressor role. In addition, an inverse correlation between ZYG11A and p53 levels in individual tumors was noticed. Taken together, our data justify further exploration of the role of ZYG11A as a novel biomarker in EOC.

## Introduction

Epithelial ovarian cancer (EOC) is the most lethal cancer among gynecological malignancies, accounting for 90% of all ovarian tumors (1, 2). Morphological, immunohistochemical and molecular genetic studies classified EOC cases into two main groups, Types I and II, on the basis of their distinctive clinico-pathological and molecular features (3). Type I tumors include low-grade serous (LGSC), low-grade endometrioid, clear cell and mucinous carcinomas. These tumors are indolent and have a relatively good prognosis, defined as stages I/II. They are characterized by a number of mutations, including aberrations in the KRAS, BRAF, ERBB2 and PTEN genes, which target specific cell signaling pathways, but very rarely p53 mutations (4). Type II tumors are more aggressive and include high-grade serous (HGSC), high-grade endometrioid, carcinosarcomas and undifferentiated carcinomas (2). Type II tumors are characterized by frequent p53 mutations, genomic instability and BRCA1 mutations (5, 6).

Approximately 80% of EOC patients are diagnosed at advanced stages when the disease has already disseminated throughout the peritoneal cavity (7). The 5-year survival rate for advanced EOC is 30%, compared to 70%-90% for early disease. Surgical cytoreduction combined with platinum-based chemotherapy and, more recently, targeted therapies have improved the survival rate for patients over the past few decades (8, 9). Unfortunately, women with platinum-resistant or refractory relapsed EOC continue to have a poor prognosis. Early detection, including identification of biomarkers, and novel effective treatments, mainly for platinum-resistant and recurrent disease, are among the critical needs in gyneco-oncology (10).

The insulin-like growth factors (IGFs) are a family of growth factors with important roles in the development and metabolism of multiple tissues and organs (11, 12). Epidemiological and clinical studies provide evidence that high endocrine IGF1 correlates with increased cancer risk (13–15). Furthermore, the IGF1 receptor (IGF1R), which mediates the biological actions of IGF1, is overexpressed in most tumors and cancer cell lines, including ovarian cancers (13, 16). This pattern of expression reflects the potent pro-survival activity of IGF1R (17). While the mechanism of action of IGF1 has been thoroughly dissected, most clinically-relevant IGF1 target genes are yet to be discovered. In recent studies, we identified the *ZYG11A* gene as a new candidate target of IGF1 (18, 19). Specifically, we demonstrated that IGF1 governs *ZYG11A* expression in endometrial cancer cells in a p53-dependent manner. The biological relevance of the IGF1-p53-ZYG11A regulatory loop remains undefined.

*ZYG11A* is a member of the *ZYG11* gene family. ZYG11A was initially identified as a cell cycle regulator (20) and, subsequently, shown to be involved in cell division during meiosis (21). Its homologue, ZYG11B, serves as a substrate recruitment subunit for a cullin-2-based E3 ubiquitin ligase (22, 23). Deregulation of this ubiquitin system is associated with a number of pathologies, including cancer. ZYG11A was recognized as a potential oncogene in non-small cell lung cancer (24). In agreement with this finding, increased ZYG11A expression in this condition was correlated with a poor prognosis. In addition, a recent study generated evidence that a missense mutation of the *ZYG11A* gene caused cell cycle arrest in pancreatic β-cells, linking malfunction of this gene to chronic hyperglycemia (25).

In view of the important roles of tumor suppressor p53 and the IGF1 axis in ovarian cancer biology and to expand our previous studies on the *ZYG11A* gene, we investigated in the present study the expression of ZYG11A, p53 and IGF1R in a collection of EOC specimens. In addition, *zyg11a* expression was assessed in ovaries and uteri of growth hormone receptor (GHR) knock-out (GHRKO) mice, an animal model of IGF1 deficiency. Data obtained revealed that (1): high ZYG11A levels correlate with low EOC histological grade (2); an inverse correlation between ZYG11A and p53 levels exists in individual tumors (3); the *zyg11a* gene is developmentally regulated, with high mRNA values detected in ovary and uteri of young mice. The association between low ZYG11A levels and high histological grade identifies the *ZYG11A* gene as a candidate tumor suppressor. The clinical implications of ZYG11A expression in EOC (and, probably, other types of cancer) merit further investigation.

## Materials And Methods

### Study Population

The study was approved by the Hillel Yaffe Medical Center, Institutional Helsinki Committee (0019-16HYMC). Formalin-fixed, paraffin-embedded (FFPE) tissue samples from 36 patients diagnosed with EOC who were operated at Hillel Yaffe Medical Center between 2012 and 2018 were obtained from the Pathology Department. Clinical and pathological data of all patients, including age, weight, pathological type and histological grade, surgical-pathological stage, treatment protocol, response to treatment, recurrent disease and survival were obtained from their medical records (26).

### Tissue Microarray (TMA) Construction and Immunohistochemistry

FFPE tissue specimens were chosen based on clinical data. Hematoxylin- and eosin-stained slides were reviewed and representative tumor samples were selected for each case. The selected area was marked for TMA construction. Out of 36 EOC cases, 29 exhibited a high-grade serous histology and seven displayed a low-grade histology. Cases were classified as follows: stage I: 8 cases; stage II: 2 cases; stage III: 15 cases; and stage IV: 11 cases. Staging was conducted according to the clinical criteria of the International Federation of Gynecology and Obstetrics (FIGO). From each case, a core of tumor tissue with a diameter size of 2 mm was punched by TMA grand master (3DHISTECH, Budapest, Hungary). FFPE TMA blocks were cut into 4 μm sections on coated slides and deparaffinized. Peroxidase was blocked using 3% H_2_O_2_. Antigens were retrieved using 1M citrate buffer for 20 min. The following primary antibodies were used: ZYG11A (#177696, Abcam plc, Cambridge, UK), IGF1R (sc-81167, Santa Cruz Biotechnology Inc., Dallas, TX, USA) and p53 (M7001, DAKO, Glostrup, Denmark). Slides were then counterstained with hematoxylin and mounted. Digital slides were scanned using Panoramic MIDI (TMA Scanner), and the images were captured using the 3DHISTECH software. Results of all samples were quantified by an experienced pathologist at the Pathology Institute, Haemek Medical Center (Afula, Israel), who was blinded to clinical data regarding the patients and samples.

### Scoring

Levels of staining were subjectively graded by the pathologist based on the number of reactive *versus* total cells (extensiveness). Immunohistochemical results of ZYG11A, p53 and IGF1R stainings were divided into low and high extensiveness groups, where the percentage of ZYG11A, p53 and IGF1R was measured using the median value. If the cell density was above the median values of 10%, 50% and 85%, respectively, the sample was defined as high staining.

### Animal Studies

The generation of the GHRKO mouse model was previously described (27). Mice were in the C57BL/6J (B6) genetic background. Weaned mice were housed 2–5 animals per cage in a facility with 12-hr light:dark cycles and free access to food and water. Animals were fed a standard mouse chow. The analyses were performed in young (7-months old) and 20-months old female mice. All animal procedures were approved by the Institutional Animal Care and Use Committee of the NYU School of Medicine (Assurance number A3435-01, USDA license No. 465), and conform to the Animal Research: Reporting of *In Vivo* Experiments (ARRIVE) guidelines (http://www.nc3rs.org.uk/arrive-guidelines). RNA from uteri and ovarian tissues was extracted with an RNAeasy plus Mini Kit (Qiagen, Hilden, Germany). Approximately 30 mg of tissue sample was homogenized with a Tissue lyser II at 30,000 rpm for 9 min. The homogenized tissues were then applied to gDNA Eliminator Mini Spin Columns and washed extensively with the buffers supplied before eluting RNA in water. Finally, 30 μl of DEPC-dH2O was added to the eluted RNA. For cDNA synthesis, 1 ug of RNA was used as per instructions in the kit (Superscript III First strand, ThermoFisher Scientific, Waltham, MA, USA). Quantitative PCR (qPCR) was performed using the following primers: *zyg11a*, forward: 5’-GTGGCCTTGAGTCATTTCACT-3’, reverse: 5’-CCAGGTTCGGTAACTGAGAAAC-3’; *β-actin*, forward: 5’-AGATGACCCAGATCATGTTTGAG-3’, reverse: 5’-TGGTACGACCAGAGGCATACA-3’.

### Statistical Analysis

Descriptive statistics in terms of mean, SD, median, percentiles and ranges were performed to the whole parameters in the study. McNemar-Bowker test and Marginal Homogeneity test was used to test the similarity for the categorical levels of the proteins. Differences between groups (high grade serous *vs*. low grade, neoadjuvant *vs*. chemo, and stage 1 + 2 *vs*. 3 + 4) for the proteins extensity were tested by Mann Whitney U test. Spearman’s correlation was used to test the relation between proteins. P < 0.05 was considered as significant. SPSS version 25 was used for the statistical analysis.

## Results

### Patients’ Baseline Characteristics

**Table 1** summarizes the baseline demographic and clinico-pathological characteristics of 36 patients diagnosed with EOC. Patients’ ages ranged between 27 to 87-years, with a mean of 62.8 ± 14 years. Ten patients had early stage disease (Stage I-II) and 26 (72.3%) had advanced stage disease (Stage III-IV). All patients had histological subtypes consistent with ovarian serous carcinoma: 7 low grade (19%) and 29 high grade (81%). The follow-up period ranged from 13 to 78 months (median = 30 months). The median progression-free survival (PFS) was 24 months (range = 7-36) and the overall survival (OS) was 38 months (range = 15-82).

**Table 1:** Demographic and pathological data of 36 patients with EOC.

Age, mean ± SD [range]	62.8 ± 14.0; [27-87]
Ethnicity mother; n=22	
Jewish	17 (77%)
Arab	5 (23%)
BMI (n=33), mean ± SD (range)	27.8 ± 6.1; [18.3-42.2]
Parity	2.83 ± 1.72; [0-8]
Comorbidities	
Hypertension	14 (39%)
Diabetes	7 (19%)
Dyslipidemia	8 (22%)
Other	22 (64%)
Primary site	
Ovary	33 (91.6%)
Fallopian tube	1 (2.77%)
Primary peritoneal	3 (8.33%)
Unknown	1 (2.77%)
Histology	
High grade serous carcinoma	29 (81%)
Low grade serous carcinoma	7 (19%)
Stage	
I	8 (22%)
II	2 (6%)
III	15 (42%)
IV	11 (31%)
Other malignancy	
Breast	2 (5.5%)
Lymphoma	2 (5.5%)
None	32 (89%)
Neoadjuvant chemotherapy	17 (47.3%)
Primary surgery	19 (52.7%)

### ZYG11A, p53 and IGF1R Expression in EOC

**Figure 1:**
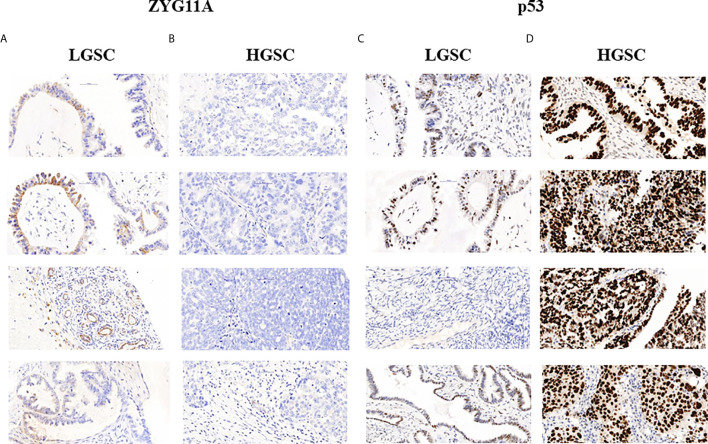
Immunohistochemical staining of ZYG11A and p53 in EOC patients. Immunohistochemical analysis of ZYG11A **(A, B)** and p53 **(C, D)** was conducted in seven patients with low grade serous carcinoma **(A, C)** and 29 patients with high grade serous carcinoma **(B, D)** (x40). TMA analyses were conducted as described in *Materials and Methods*. The figure shows the images of four representative LGSC and HSGC patients.

**Figure 2:**
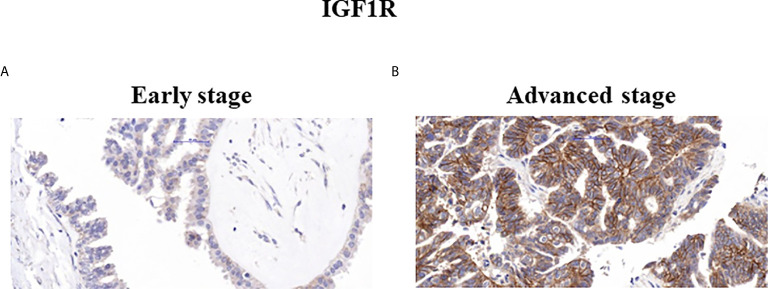
Immunohistochemical staining of IGF1R. Representative samples of IGF1R staining in early **(A)** and advanced **(B)** ovarian serous carcinoma patients (x40).

The levels of expression of ZYG11A, p53 and IGF1R proteins are summarized in **Table 2**. High staining of ZYG11A was seen in 22.2% of EOC cases whereas an opposite pattern of expression was exhibited by p53 and IGF1R. Thus, enhanced p53 and IGF1R immunoreactivities were demonstrated in 80.6% and 66.7% of cases, respectively. Typical immunohistochemical stainings of ZYG11A, p53 and IGF1R are presented in **Figures 1**, **2**.

**Table 2:** Immunohistochemical staining rates in EOC tissue.

Tissue samples	n=36	Proportion (%)
IGF1R		
Low stainin	12	33.3
High staining	24	66.7
P53		
Low staining	7	19.4
High staining	29	80.6
ZYG11A		
Low staining	28	77.7
High staining	8	22.2

### Correlation Between Immunohistochemical Staining and Clinico-pathological Characteristics

Mann Whitney U test revealed that ZYG11A and p53 stainings significantly correlated with histological grade (P = 0.011 and P = 0.018, respectively) (**Table 3**). Thus, high ZYG11A levels were associated with low grade (LGSC) and p53 levels correlated with high grade (HGSC) EOC. Nevertheless, there was no correlation between ZYG11A and p53 with pathological stage. Moreover, TMA analysis demonstrated high (P = 0.05) IGF1R intensity at advanced EOC stages in comparison to early stages (**Figure 2** and **Table 3**). However, no correlation was seen between IGF1R and histological grade (P = 0.19). In addition, no correlation was seen between ZYG11A, p53 and IGF1R expression with PFS (P = 0.357, 0.491 and 0.369, respectively) or OS (P = 0.96, 0.76 and 0.096, respectively).

**Table 3:** Correlation between immunohistochemical staining and clinico-pathological parameters.

Pathological data	STAGE		GRADE	
	I+II	III+IV	Low	High
**IGFIR**				
Low	6 (60%)	6 (23.1%)	4 (57.1%)	8 (27.6%)
High	4 (40%)	20 (76.9%)*	3 (42.9%)	21 (72.4%)
**p53**				
Low	1 (10%)	6 (23.1%)	2 (28.6%)	5 (17.2%)
High	9 (90%)	20 (76.9%)	5 (71.4%)	24 (82.8%)*
**ZYG11A**				
Low	5 (50%)	23 (82.1%)	3 (42.9%)	25 (86.2%)
High	5 (50%)	3 (17.9%)	4 (57.1%)	4 (13.8%)*

*P ≤ 0.05.

### Correlation Between ZYG11A and p53 Expression Levels

**Figure 3:**
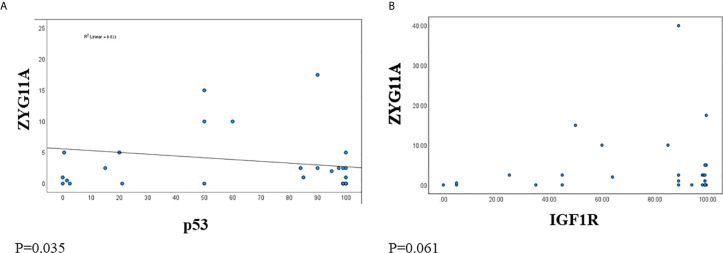
Correlation between ZYG11A, p53 and IGF1R expression in EOC. Pearson correlation analysis was conducted between ZYG11A and p53 expression values **(A)** and between IGF1R and ZYG11A values **(B)** in individual EOC patients (P < 0.05).

Next, we analyzed the correlation between ZYG11A and p53 expression levels in EOC patients. A significant negative correlation was observed between ZYG11A and p53 values (**Figure 3A**). This was reflected by a strong Pearson correlation (*r* = 0.021, P = 0.0355). On the other hand, no significant correlation was seen between ZYG11A and IGF1R protein levels (P = 0.061) (**Figure 3B**).

### Animal Studies

**Figure 4:**
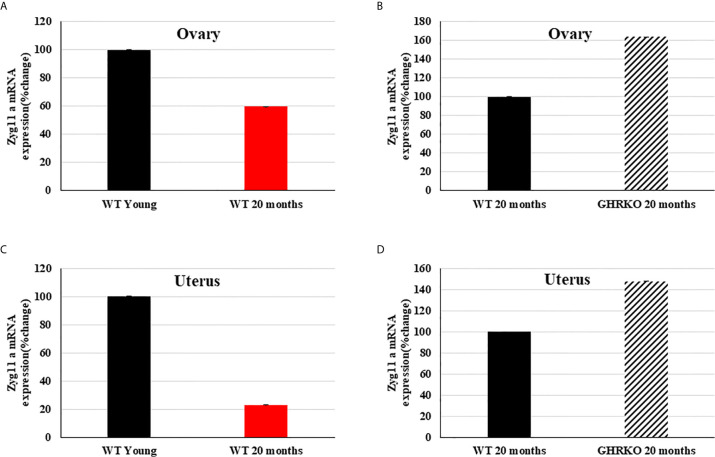
Expression of zyg11a mRNA in ovaries and uteri of GHRKO and WT mice. Ovaries and uteri tissue of young (7-months old) and 20-month old WT mice and 20-month old GHRKO mice were obtained and total RNA was prepared as described in Materials and Methods. Zyg11a mRNA levels were measured by qPCR and normalized to the corresponding β-actin mRNA values. Bars represent mean ± SD of 4-5 animals. **(A, C)** a value of 100% was given to the zyg11a mRNA values in young mice. **(B, D)** a value of 100% was given to the zyg11a mRNA values in 20-month old WT mice. Differences between groups in all four graphs were highly significant (P<0.01). When not shown, error bars were smaller than the symbol size.

Finally, we investigated the developmental and IGF1-stimulated regulation of the *zyg11a* gene in mouse uteri and ovaries. To this end, the mRNA expression profile of *zyg11a* was determined in young (7-months old) and 20-months old WT mice and in 20-months old GHRKO animals by qPCR. In both organs, zyg11a mRNA levels were significantly decreased (40% and 77% reductions in ovary and uteri, respectively) in old, compared to young, WT animals (**Figures 4A, C**). GHRKO mice exhibited enhanced (64% and 48% increases in ovary and uteri, respectively) zyg11a mRNA levels compared to WT animals (**Figures 4B, D**). Taken together, data is consistent with an ontogenetic reduction in *zyg11a* gene expression in both organs. In addition, elevated zyg11a mRNA levels in tissues of GHRKO mice, an animal model associated with igf1 deficiency, confirms human data showing that ZYG11A is a target for negative regulation by IGF1 (18, 19).

## Discussion

The identification of biomarkers that can help in the diagnosis, classification and prognosis assessment of EOC patients is of cardinal importance in the area of gynecological cancers (9, 28). The contribution of environmental factors, including obesity and a sedentary lifestyle, to the development and progression of this group of malignancies has been the focus of major interest in recent years (29–31). The role of the IGF1 signaling axis on cancer etiology has been well established (32, 33). Special interest on this family of growth factors, receptors and binding proteins stems from the fact that the IGFs play key cellular and biochemical roles in the chain of events responsible for the acquisition of a transformed phenotype (34–36). Of clinical relevance, the IGF1R emerged as a promising therapeutic target in oncology (37–40). Unfortunately, most clinical trials conducted to date led to disappointing results that, in a large portion of the cases, were due to lack of biomarkers capable of identifying patients who might benefit from this type of intervention (41).

In the specific context of ovary, IGF1 and the IGF1R play important roles in regulating the normal biology of ovarian surface epithelial cells and have also been implicated in the transformed phenotype of EOC cells (28). It was reported that EOC cells display an autocrine growth loop mediated through the IGF1R (42). The impact of endocrine IGF1 on EOC development remains controversial. Whereas prospective studies reported a significant correlation between serum IGF1 levels and EOC risk among women younger than 55-years (43), other studies showed decreased serum IGF1 concentrations in postmenopausal patients with ovarian tumors (44).

Recent genomic analyses of Laron syndrome (LS) patients, a type of dwarfism under the spectrum of the congenital IGF1 deficiencies, allowed us to identify *ZYG11A* as a new downstream target for IGF1 action (18). Specifically, we generated evidence that ZYG11A mRNA levels were 3-fold higher in LS-derived lymphoblastoid cells than in healthy controls. Given the epidemiological data showing that LS patients are protected from cancer development (45), we hypothesized that (1): the *ZYG11A* gene might be negatively regulated by ambient IGF1 levels; and (2) ZYG11A might fulfill a protective, anti-proliferative role, at least in certain types of cancer.

The present study was designed to evaluate the expression of ZYG11A in a collection of EOC samples and to explore potential connections between ZYG11A levels, histological grade and pathological stage. Furthermore, we assessed the correlations between ZYG11A expression with that of tumor suppressor p53 and the IGF1R. The rationale for this search was the recent finding that IGF1 was able to inhibit ZYG11A mRNA and protein levels in endometrial cancer-derived cells containing a wild-type, but not a mutant, *p53* gene (19). On the other hand, IGF1 potently stimulated ZYG11A expression in mutant *p53*-expressing cells. While the clinical implications of the IGF1-p53-ZYG11A regulatory loop remain unsettled, we believe that elucidation of the functional interplay between these gene products might be of importance in the cell’s decision whether to adopt proliferative or apoptotic paths.

Our immunohistochemical analysis provides evidence that low ZYG11A levels were associated with high EOC histological grade. This pattern of expression is consistent with a candidate tumor suppressor role of ZYG11A, at least in the context of ovarian tumors. On the other hand and probably as a result of the small size of our study (n=36), we found no correlation between ZYG11A expression with PFS and OS. In addition, we report a negative correlation between ZYG11A and p53 expression levels in EOC. While the mutational status of p53 in our collection has not been assessed, mutation of this gene constitutes the most common genetic event in sporadic EOC (46). The majority of high grade serous ovarian carcinoma harbor inactive p53 molecules that are unable to prevent the development and progression of tumors. Furthermore, a high rate of p53 mutations at advanced cancer stages may explain the enhanced IGF1R expression at these pathological stages (47). Finally, comprehensive molecular analyses provide evidence that the IGF1 and p53 signaling pathways converge at both physical and functional levels (48).

Early studies have identified ZYG11A as a potential oncogene in non-small cell lung cancer (24). These studies were based on the Cancer Genome Atlas database and demonstrated that ZYG11A was highly expressed in tumor, compared to adjacent, lung tissue. In addition, the authors indicate that ZYG11A expression correlated with a poor prognosis. These studies are in line with our previous results showing that ZYG11A silencing in endometrial cancer cells led to a marked decrease in proliferation and increase in the proportion of apoptotic cells (19). Furthermore, ZYG11A was shown to be involved in cell cycle regulation as well as in the expression of a series of classical cell cycle regulatory genes. Thus, abrogation of ZYG11A expression led to a decrease in cyclin D1 levels and enhanced p53 and p21 concentrations. Likewise, ZYG11A was shown to function as a cell cycle regulator required for β-cell growth (25). ZYG11A-deficient β-cells were arrested at the G2/M phase of the cell cycle, leading to a drastic reduction in growth rate. The finding that high histological grade EOC exhibit low ZYG11A amounts is consistent with a protective, anti-oncogenic role of this protein. Future studies will be aimed at characterizing the tissue-specific range of activities of ZYG11A.

Finally, animal studies were conducted in young and 20-month old mice in order to investigate a potential developmental regulation of *zyg11a* gene expression. These analyses detected a marked decrease in zyg11a mRNA levels in uteri and ovaries of older, compared to young, mice. Given the fact that IGF1 levels are up-regulated in older animals (49, 50) and in view of our results showing negative regulation of ZYG11A by IGF1, data is consistent with an IGF1-dependent down-regulation of zyg11a tissue levels throughout development. The direct role of the GH/IGF1 axis in regulation of *zyg11a* gene expression was investigated using the GHRKO mouse model. Consistent with the human data, zyg11a levels were markedly increased in conditions associated with IGF1 deficiency (18).

In summary, we have identified the *ZYG11A* gene as a potential biomarker in EOC. Our analyses provide evidence of reduced expression of ZYG11A in high grade tumors, consistent with a putative tumor suppressor role. Finally, the clinico-pathological relevance of the present study must be further investigated.

## Data Availability Statement

The raw data supporting the conclusions of this article will be made available by the authors, without undue reservation.

## Ethics Statement

The studies involving human participants were reviewed and approved by Hillel Yaffe Medical Center Helsinki Committee. The patients/participants provided their written informed consent to participate in this study. The animal study was reviewed and approved by Institutional Animal Care and Use Committee NYU. Written informed consent was obtained from the owners for the participation of their animals in this study.

## Author Contributions

LA, LS-G, HW, and IB conceived of and designed the study. The experimental procedures were performed by LA, LS-G, SM-S, RS, and MD. Data analysis and interpretation was conducted by LA, LS-G, SY, MH, ZL, HW, and IB. Statistical analyses were conducted by LS-G and IB. HW and IB prepared the manuscript. All authors contributed to the article and approved the submitted version.

## Funding

This research was funded by the Israel Cancer Research Foundation, Montreal, Canada (Grant number 2026011), and The Ruth and Bruce Rappaport Faculty of Medicine, Technion, Israel Institute of Technology, Haifa, Israel, to IB.

## Conflict of Interest

The authors declare that the research was conducted in the absence of any commercial or financial relationships that could be construed as a potential conflict of interest.
